# Big Data Analytics to Reduce Preventable Hospitalizations—Using Real-World Data to Predict Ambulatory Care-Sensitive Conditions

**DOI:** 10.3390/ijerph20064693

**Published:** 2023-03-07

**Authors:** Timo Schulte, Tillmann Wurz, Oliver Groene, Sabine Bohnet-Joschko

**Affiliations:** 1 Faculty of Management, Economics and Society, Witten/Herdecke University, 58455 Witten, Germany; 2 Faculty of Health, Witten/Herdecke University, 58455 Witten, Germany; 3 Department of Business Analytics, Clinics of Maerkischer Kreis, 58515 Luedenscheid, Germany; 4 Department of Project and Change Management, University Clinic Hamburg-Eppendorf, 20251 Hamburg, Germany; 5 Department of Research & Innovation, OptiMedis AG, 20095 Hamburg, Germany

**Keywords:** real-world evidence, prediction model, claims data, machine learning, integrated care, ambulatory care-sensitive conditions, hospitalization, prevention, population health

## Abstract

The purpose of this study was to develop a prediction model to identify individuals and populations with a high risk of being hospitalized due to an ambulatory care-sensitive condition who might benefit from preventative actions or tailored treatment options to avoid subsequent hospital admission. A rate of 4.8% of all individuals observed had an ambulatory care-sensitive hospitalization in 2019 and 6389.3 hospital cases per 100,000 individuals could be observed. Based on real-world claims data, the predictive performance was compared between a machine learning model (Random Forest) and a statistical logistic regression model. One result was that both models achieve a generally comparable performance with c-values above 0.75, whereas the Random Forest model reached slightly higher c-values. The prediction models developed in this study reached c-values comparable to existing study results of prediction models for (avoidable) hospitalization from the literature. The prediction models were designed in such a way that they can support integrated care or public and population health interventions with little effort with an additional risk assessment tool in the case of availability of claims data. For the regions analyzed, the logistic regression revealed that switching to a higher age class or to a higher level of long-term care and unit from prior hospitalizations (all-cause and due to an ambulatory care-sensitive condition) increases the odds of having an ambulatory care-sensitive hospitalization in the upcoming year. This is also true for patients with prior diagnoses from the diagnosis groups of maternal disorders related to pregnancy, mental disorders due to alcohol/opioids, alcoholic liver disease and certain diseases of the circulatory system. Further model refinement activities and the integration of additional data, such as behavioral, social or environmental data would improve both model performance and the individual risk scores. The implementation of risk scores identifying populations potentially benefitting from public health and population health activities would be the next step to enable an evaluation of whether ambulatory care-sensitive hospitalizations can be prevented.

## 1. Introduction

Health systems in developed countries face a variety of challenges, including a rising demand for health services due to demographic changes, increasing multi-morbidity, unhealthy behaviors and financial constraints [1]. These challenges are reinforced by highly fragmented processes of healthcare delivery, which may be overcome in care models and settings that focus on creating value for individuals and also incorporate preventative action [2]. Putting people rather than siloed provider structures or diseases in the center, integrated health systems are fueled by an integration of health information technology infrastructure and can benefit from advanced models of health data analytics [3,4]. Big data analytical capabilities are recognized as one of the most important innovations in healthcare in the recent decade [5,6], and advances in prediction models provide great opportunities, e.g., in the identification of risk groups or in the prediction of hospitalization. One field of specific political interest is the analysis and reduction of ambulatory care-sensitive hospitalizations (ACSH), i.e., inpatient hospital cases that are at least partly considered avoidable with improved care in the outpatient sector in the context of nursing homes or through prevention achieved, e.g., by public health activities [7,8,9]. Reductions in ACSH can both improve the patient experience and avoid an unnecessary usage of health system resources so that the ACSH-rate is also used as a measure of healthcare quality [10,11]. A study analyzing the cost associated with ACSH in the German health insurance system estimated a cost of EUR 3.5 billion per year (increasing per year by 0.9%) based on the mean costs of such hospital cases from the German Diagnosis Related Group (DRG) system [12].

To support action towards reduction of unnecessary hospital cases, the aim of this study was to develop a prediction model based on real-world claims data to identify individuals or populations with a high risk of being hospitalized due to an ambulatory care-sensitive condition who then might get special attention or benefit from tailored prevention activities or treatment options. This is comparable to an approach of the Veterans Health Administration providing patient-specific care assessment need scores based on data from the corporate data warehouse that can be accessed by healthcare providers and population health managers [13]. Several studies exist predicting (re-)hospitalizations in general [7,14,15,16,17,18,19], but only a few specifically predict ACSH in the context of the health systems of the USA, Canada and Italy [10,20,21,22]. While the methodologies are comparable to a certain extent, this study extends the context to Germany, which is on the one hand valuable since ACSH definitions are most often adapted to the specific health system characteristics and therefore models and results from other contexts cannot be directly transferred or put into practice. On the other hand, just the fact that the results of this model are actually implemented in regional population health and integrated care interventions in Germany is another special feature of this work. Based on risk scores and predefined thresholds, warning signs could be implemented in the information systems of responsible medical and non-medical experts who can then suggest certain measures or adjust their actions. The action derived from such risk assessments would ideally lead to improved prevention, better healthcare quality for those affected by or being at risk of certain diseases and reduced cost for the community [23]. To achieve a reliable prediction, a statistical model based on a logistic regression was compared to a machine learning model based on the Random Forest method.

## 2. Materials and Methods

In the following section, the concept of ambulatory care-sensitive hospitalizations is described followed by a description of the database and the analytical method of model construction. The section closes with a definition of the outcome variable and the independent variables of the prediction models.

### 2.1. Ambulatory-Care Sensitive Conditions/Hospitalizations

Due to inconsistent definitions and varying national health system characteristics, there is no scientific consensus on which conditions are understood to be ambulatory care-sensitive conditions (ACSC) or what defines an ambulatory care-sensitive hospitalization (ACSH). Generally, an ACSC is a diagnosis for which timely and effective activities “can help to reduce the risk of hospitalization by either preventing the onset of an illness or condition, controlling an acute episodic illness or condition, or managing a chronic disease or condition”, and an ACSH is a hospitalization due to an ambulatory care-sensitive condition [7]. The international statistical classification of diseases and related health problems (ICD) helps to make definitions comparable but coding and care provision may differ at the regional or country level [24]. Due to specific health system characteristics, some diseases might be treated as inpatient cases in one context and as ambulatory cases in another. Since this study is built on German data, a definition developed for the German healthcare system was used. This categorization of ACSC contains 258 singular ICD-10 diagnoses, summarized in 40 groups of which 22 groups constitute a core list. The 22 groups of the core list have a relatively high preventability score of more than 50%, varying between 58% for gonarthrosis and 94% for dental diseases [8]. See Sundmacher et al. for the full list of ICD-10 codes of ambulatory-care-sensitive conditions used for this study [25]. At the least, the core list includes chronic diseases that are also commonly included in definitions of ACSC in the context of other countries [10].

### 2.2. Database

The database used for this study is deidentified insured-level claims data (*n* = 69,392) from two regional integrated care networks of OptiMedis AG, an integrated care management organization [26]. The regions are set as one rural and one urban area, each accounting for nearly half of the population size. Data were fully available for the years 2016–2019. The data set itself does not fulfil the 3-V characteristics of big data [27,28]. However, it has been shown that claims data are valuable in assessing quality and efficiency of care and have the advantage of being easily accessible in an electronic format without needing additional documentation [29]. The database contains information on patient demographics, in- and outpatient care, work incapacity, drugs, nonmedicinal remedies and aids, rehabilitation and long-term care services [30]. To account for country specifics in the data, the German guideline for claims data analysis was considered [31].

### 2.3. Big Data Analytics and Prediction Models

For big data analytics, there is also no agreed-upon definition. Performing predictive or explorative analytics (taken together also labelled as advanced analytics) on sets meeting the definition of big data is one approach to define big data analytics [5,6]. Another refers to the usage of inductive machine learning approaches suited for high-dimensional data sets [32]. As the database available for this study did not fulfil the 3-V characteristics, the second definition is adapted, and the term big data analytics therefore refers to the method instead. Most of the models in the literature rely on statistical methods, especially the logistic regression, and machine learning methods, such as Random Forests, Neural Networks or Support Vector Machines [14,15]. In this study, the predictive performance of a statistical model (logistic regression) is compared to that of a machine learning model (Random Forest). Supervised machine learning, such as the Random Forest model, is flexibly applicable on complex data of various structures. During the model building process, assumptions about the data distribution can be adapted, whereas most Random Forest algorithms assume a Gaussian distribution per default. Furthermore, the outcome variable has to be human-labelled, and the prediction is deduced based on three stages in a causal chain: training, validation and testing [33,34]. To train the model, a data set is analyzed to identify discriminating features of the predictor and optimization algorithms are performed to reproduce the outcome [35]. The Random Forest model randomly selects a predefined number of distribution criteria and grows several trees that categorize the individual observations. A majority vote over all trees then defines the class. There is not one specific Random Forest algorithm, rather many different algorithms exist. This analysis was performed in R statistics using the ranger package [36]. The number of variables tested at each node was the square root of the number of numerical variables. The number of iterations, i.e., the number of trees in the forest, was set to 500 [37].

### 2.4. Outcome Variable and Independent Variables

The outcome variable was defined similar to prior studies focusing on ACSH prediction [10,20,21]. It is the event of an individual being hospitalized with an ACSC in the prediction year. The full list model of ACSC comprises the above-mentioned 258 singular ICD-10 diagnoses. To assess whether it improves the model performance, an outcome variable was also defined, focusing only on the core list of ACSC with only 164 diagnoses (core list model) [8]. Death was not investigated as no information regarding the cause of death was available.

Independent variables with a high predictive value in previous studies were medical diagnoses and prescribed medications, prior healthcare utilization as well as multimorbidity and polypharmacy measures [16]. The following variables were used for the construction of the prediction models: age as a categorical variable in 16 classes (0–14, 15–19, 20–24, 25–29, 30–34, 35–39, 40–44, 45–49, 50–54, 55–59, 60–64, 65–69, 70–74, 75–79, 80–84, ≥85), gender (male vs. female), insurance status (employees, pensioners, children, unemployed, others), number of physician visits (GP and specialists), days of incapacity for work, number of hospitalizations (all-cause and ACSH); length of hospital stays in days, mean number of drug prescriptions per quarter (drug count), a polypharmacy measure (max amount prescribed on a given day), a multimorbidity measure (modified Charlson score [38]), enrollment in a German disease management program (coronary heart disease, asthma, type 2 diabetes, COPD), long-term care level (categorical variable in 4 classes: 0 = no care level, 1 = lowest care level, 2 = medium care level and 3 = highest care level including special hardship cases), days in any long-term care level (except 0) per year (0–365) and an inpatient and outpatient medical disease history of ACSC (distinct ACSC groups based on the International Statistical Classification Of Diseases And Related Health Problems, 10th revision, German Modification, discharge diagnoses in the inpatient setting and diagnoses with the feature “ensured” in the outpatient setting). All variables cover a time horizon of four years.

## 3. Results

### 3.1. Model Construction and Descriptive Cohort Analysis

The process of model construction distinguishes between training and test data sets. In this study, 2019 was set as the prediction year. Thus, model building was conducted on a training set from 2018, whereas the disease history was observed from 2016 to 2018. Model evaluation was performed based on the test set with the outcomes being observed in 2019. The exclusion of certain variables is a common step in designing risk prediction models. The insurance duration was a major exclusion criterion. In order not to include individuals that were not insured with their current health insurance company for a considerable amount of time and thus had missing data, a threshold was determined. Individuals had to be insured for 360 days or more in the prediction year as well as for at least 300 days in each of the previous four years. Thereby, deceased individuals were indirectly excluded which was considered as unproblematic as it is doubtful whether the respective hospital cases might have been preventable in the sense of the ACSH concept. Of the list of ACSC, the group “rare diseases with 5000 cases each” was also excluded as not enough cases were documented in the data set.

To better understand the characteristics of the population with an ACSH, descriptive analyses of the underlying demographics were performed. Results are presented in Table 1. A rate of 4.8% of all individuals had an ACSH in 2019, and 6389.3 hospital cases per 100,000 individuals could be observed. As expected, the population with an ACSH is older, has a higher comorbidity score and higher utilization measures in nearly all sectors.

Table 2 displays the ACSH cases from the core list (22 diagnosis groups) per 100,000 individuals in the prediction year 2019. The most common ACSC disease groups in the study population were cardiovascular diseases, bronchitis and chronic obstructive pulmonary disease (COPD), mental disorders and infectious diseases.

Independent variables with a significant effect on the outcome prediction for having an ACSH in the subsequent year according to the logistic regression models are displayed in Table 3. See Table A1 and Table A2 in Appendix A for the regression coefficients, odds ratio (OR) and confidence intervals (CI; 95%) of all variables of the logistic regressions. A significant negative correlation with an odds ratio below 1 was found for being female and having an outpatient diagnosis for diseases of the skin. The latter finding might be due to the fact that these conditions in the regions observed are treated most often in an outpatient setting. Besides switching to a higher age-class, which has a strong positive correlation, the strongest feature for having an ACSH was having a previous outpatient diagnosis from the disease group “maternal disorders related to pregnancy”, pointing to the fact that expectant mothers with health problems during their pregnancy take advantage of hospital care at an above average rate and thereby have an increased risk of subsequently receiving a discharge diagnosis included on the ACSC list. The birth itself or related complications during birth are of course not part of the ACSC list. Further significant positive correlations were found for switching to a higher level of long-term care, a unit increase in the number of prior hospitalizations (all-cause and cases due to an ACSC), and unit increases of the drug count and the number of specialist visits. Specific previously documented disease groups with a significant effect were, e.g., alcohol-related disorders, circulatory diseases, ear nose throat infections and diabetes in the outpatient setting, heart failure and hypertension in the inpatient setting or depressive disorders in both settings. Due to the rather small number of persons with long-term care and sick leaves in the sample, small but significant effects were also found for a unit increase (numerical variables ranging from 0–365) of the days in a high long-term care level or a unit increase of the duration of sick leaves in days. Having a diagnosis of heart failure was only significant in the core list model. Quite surprisingly, the number of GP visits did not show a significant effect. The fact that the Charlson comorbidity score did not show a significant effect with ACSH might be because this index was originally developed to predict one-year-mortality rates in hospital [39] so that the conditions taken into consideration might be severe rather than preventable as defined by the ACSH concept.

With respect to the Random Forests, variable importance values were calculated using the impurity-corrected mode based on the Gini Index as part of the ranger package [36]. In the core list scenario, drug count, previous hospitalizations (all-cause, due to an ACSC, due to diabetes or due to hypertension) and the duration of a hospital stay in the previous year were the variables with the highest predictive value.

### 3.2. Comparison of the Predictive Model Performances

The performance of the models was evaluated and compared based on the c-statistics. The c-statistics point to the fact that the Random Forest model performs slightly better than the logistic regression model in predicting the outcome variable of having an ACSH in the prediction year, both in the full list and in the core list scenario (see Table 4).

For a subset of the data from one health insurance company (*n* = 29,275), further evaluation criteria in the form of sensitivity, specificity and the positive and negative predictive value were applied [23]. Related to the outcome variable, sensitivity is defined as the percentage of individuals with an ACSH that are correctly identified as having an ACSH in the upcoming year. Specificity, on the other hand, relates to the number of individuals without an ACSH that are identified as such. Additional risk thresholds also used by Louis et al. [21] were implemented. The category “high risk” includes individuals with a predicted probability of 15% to 24%; the category “very high risk” includes individuals with a predicted probability of 25% and higher to have an ACSH in the prediction year. For the core list scenario, this categorization results in the values summarized in Table 5. Generally speaking, for these two cut-off points, the Random Forest achieved higher sensitivity scores but lower specificity scores, i.e., from the very high risk cohort it identifies more individuals who actually have an ACSH in the upcoming year than the logistic regression (50.0% versus 42.9% for the core list model). However, it also identifies more individuals erroneously (1 minus the specificity, i.e., 11.1% versus 8.9% of the population not having an ACSH). Vice versa, the positive predictive value for the logistic regression is higher.

## 4. Discussion

In the course of efforts to improve value in health systems, tools such as prediction models for ACSH can provide a valuable contribution to better steer interventions and allocate resources. In this paper, a risk prediction model with good reliability and wide applicability based on routinely collected administrative data was developed that can be used to improve not only primary care but also population health management and public health prevention by supporting providers with additional information. The fact that age has a strong positive correlation with ACSH is in line, e.g., with a population-based analysis of ACSC in Ireland showing that 69.1% of all ACSCs were found in adults over 65 [40]. The diagnosis groups with a high odds ratio, such as maternal disorders related to pregnancy, mental disorders due to alcohol or opioids, alcoholic liver diseases, certain diseases of the circulatory system or depressive disorders, could give hints for population health managers about which risk groups to address with intensified effort in a region. The individually calculated risk scores could be implemented in clinical or non-clinical information systems within the integrated care systems as an extension of the information base of the providers. Conversely, if further data, e.g., extracted directly from electronic health records, were also incorporated into the prediction models, not only more accurate, but also more up-to-date results could be calculated.

The models developed in this publication achieved c-statistics comparable to Billings et al. (0.780) [7] and Yi et al. (0.805) [10], indicating a good model fit above the median of 0.68 of a systematic review of prediction models for rehospitalization [10]. However, perhaps due to the smaller sample size, the model performance did not reach that of Louis et al. (0.856) [21] or Gao et al. (0.833) [20]. In contrast to other studies in the field of hospital care, in this study we did not discriminate between emergency and elective admissions following the argument that an elective inpatient episode can also be a sign of unforeseen deterioration. One special feature in this study is that the Random Forest model outperforms the logistic regression model in both scenarios. The differences are not very pronounced and seem to decrease when the ACSC diagnoses are specified via the core list. Although reaching slightly higher c-values, a substantial benefit of the machine learning technique over the logistic regression model could not be found. In this specific use case, this might have been due to the fact that the database did not meet the 3-V criteria of big data. It seems understandable that a machine learning methodology alone does not lead to a superior outcome prediction because such methods applied to rather small data sources are limited in their ability to optimize the inductive feature selection process they are designed for [41,42]. Compared to a statistical regression model, it is more difficult for a machine learning model, such as Random Forest, to elucidate why one independent variable is more important than another in the feature selection process. While this may be negligible in a result-oriented perspective of calculating individualized risk scores, a link to causality and deliberations about the meaningfulness of the results should nevertheless be part of a comprehensive data mining approach [41]. Aspiring to the task of supporting providers with additional information on risk groups, in this regional context there seems to be no clear advantage of the Random Forest model. In general to date, big data analytics in healthcare found little evidence of anything surprisingly new that can effectively improve decision making or medical outcomes [43]. This does not mean that such methods do not have the potential to do so. Rather, data exchange and people-centered data collection may need to be further developed first [4]. Although predictions were meant to be derived for people in the context of the integrated care systems so that training and test sets contained the same persons, it might be valuable to test the predictive performance in populations which were not part of the training set, which was not possible in this context due to limited data availability.

A general limitation with respect to claims data is that it is collected for billing purposes, rendering it vulnerable to changes in the remuneration system, specific coding schemes or documentation errors, thus affecting the prediction results [44]. In addition, the decision of which ACSC to consider in the model building process affects the results, hampers cross-country comparisons and should be part of an ongoing model refinement process. Model refinement activities, such as hyperparameter tuning, would be useful extensions which were not applied in this study as a split of the training set into various subsets would most likely have led to subsets being too small for cross validation. Generally, most prediction models would likely benefit if a bigger data set and more independent variables were available for model optimization. Potentially valuable variables not covered in claims data would be, e.g., specific medications and dosages, ethnicity, marital status, behavioral data, lab test results, environmental data such as pollution or neighborhood characteristics, information on social support, living arrangements, the availability and proximity of hospitals as well as ambulatory treatment options [45,46], socioeconomic data, biomarker data, data from health sensors or patient-reported (outcome) data [4]. However, if additional data were to be integrated, other challenges such as interoperability would likely occur [47]. Usage of data directly extracted from primary systems, such as electronic health records, or from health platforms could enable timelier predictions as claims data encompass a certain time lag due to billing procedures.

To avoid underperforming models mis-informing clinical decision makers, analytical modelling standards and an agreed-upon framework for transparent evaluation would be needed [48]. This also implicates ethical issues, e.g., if a prediction model provides seriously harmful recommendations for some individuals. This ethical concern is not applicable in the current use case because the risk scores are only meant to support public health, population health managers or clinicians in deciding additional or intensified interventions without any proposal or judgement about the different options. Nevertheless, an appropriate framework for privacy protection and patient consent is indispensable. A subsequent general challenge for prediction models and the resulting risk scores is their factual application in the daily routines of public health or clinicians [49]. From an organizational perspective, resistance against expanding electronic data exchange between different stakeholders/parties and redesigning workflows with data-driven feedback need to be overcome [13,50] so that pilot interventions seeking to reduce ACSH can have measurable effects. Transferring the model to new regions might assess how these differ from the ones analyzed in this study. In all likelihood, other disease groups or continuous variables will show significant effects, leading to adapted intervention planning and allowing a cross-regional comparison based on the same outcome definition.

## 5. Conclusions

The risk score predictions presented in this study might be a starting point for reducing the number of ACSH on a regional level within an integrated care model incorporating public and population health activities and clinical process improvements. To proactively prevent ACSH, the results of such prediction models could steer interventions to those individuals with the highest risks and support decision making for which preventative action might be appropriate to deliver the best care or who might benefit from extra attention outside of the inpatient sector. Important next steps include continuously updating and refining the model with new data. Multidisciplinary teams will be involved to build practical and feasible solutions that engage stakeholders in the care process to use the results of such models, provided that the scores prove to be reliable. Once the accuracy of the risk scores presented here has been further tested, the next question is whether it can prevent future hospital admissions or at least delay them and thus reduce the overall number of admissions. To answer this question, further studies and evaluations would be needed that focus on gaining impact with such prediction models.

## Figures and Tables

**Table 1:** Descriptive analytics of the ACSH cohort in 2019.

Variable	Individuals without ACSH (2016–2018)	Individuals with ACSH (2016–2018)
No. of insurees	66,214	3178
Mean age	49.76	67.22
Proportion of women %	49.31	50.86
Charlson Comorbidity Score	0.21	0.63
Outpatient visits per year (GP)	2.43	3.49
Outpatient visits per year (specialist)	3.10	4.92
Hospital cases per year (all-cause)	0.20	0.70
Hospital cases per year (ACSH)	0.11	0.51
No. of prescriptions per year	2.65	5.29

**Table 2:** Descriptive analytics of the ACSH cases per 100,000 individuals in 2019.

ACSH Diagnosis Group (Core List)	Cases Per 100 k Individuals ↓
Heart failure	566.8
Other diseases of the circulation system	479.7
Bronchitis andCOPD	471.8
Depressive disorders	417.6
Ischemic heart diseases	398.0
Mental/behavioral disorders due to alcohol or opioids	386.6
Influenza and pneumonia	365.3
Ear nose throat infections	267.4
Other avoidable mental and behavioral disorders	231.9
Diabetes mellitus	227.1
Gonarthrosis (arthrosis of knee)	224.5
Hypertension	218.6
Gastroenteritis and other diseases of intestines	209.4
Soft tissue disorders	202.9
Back pain (dorsopathies)	192.9
Intestinal infectious diseases	181.8
Diseases of the skin and subcutaneous tissue	170.3
Diseases of the eye	146.1
Diseases of urinary system	146.0
Sleep disorders	74.1
Malnutrition and nutritional deficiencies	56.0
Dental diseases	36.5

(**↓** arranged in descending order by cases per 100,000 individuals).

**Table 3:** Odds ratio of significant independent variables (except age classes) of the logistic regression models for predicting ACSH in the two scenarios.

Variable	Odds Ratio (95% CI *) (Full List Scenario)	Odds Ratio (95% CI *) (Core List Scenario)
Female	0.826 (0.800–0.853)	0.819 (0.773–0.847)
OCD—Diseases of the skin and subcut. tissue	0.905 (0.885–0.918)	0.900 (0.830–0.969)
OCD—Maternal disorders related to pregnancy	7.079 (4.874–10.281)	4.881 (3.930–8.291)
OCD—Mental disorders due to alcohol/opioids	2.520 (1.799–3.244)	2.706 (1.963–3.539)
OCD Alcoholic liver disease	2.418 (1.120–4.490)	2.464 (1.192–4.331)
Long-term care level (2)	1.333 (1.126–1.580)	1.338 (1.131–1.582)
Long-term care level (3)	1.328 (1.119–1.575)	1.255 (1.058–1.488)
HDD—Heart failure	1.249 (0.991–1.606)	1.244 (1.003–1.573)
HDD—Essential hypertension	1.216 (1.022–1.348)	1.173 (1.047–1.300)
OCD—Other diseases of the circulation system	1.211 (1.103–1.332)	1.295 (1.243–1.807)
No. of ACSH	1.197 (1.104–1.308)	1.199 (1.114–1.320)
No. of hospital stays	1.172 (1.125–1.244)	1.175 (1.109–1.269)
OCD—Diabetes mellitus	1.172 (1.019–1.406)	1.192 (1.114–1.551)
OCD—Ear nose throat infections	1.132 (1.058–1.173)	1.153 (1.083–1.353)
HDD—Depressive disorders	1.130 (0.943–1.306)	1.128 (1.077–1.983)
OCD—Depressive disorders	1.104 (1.016–1.213)	1.098 (1.041–1.249)
Drug count	1.050 (1.036–1.064)	1.059 (1.016–1.239)
No. of outpatient visits (specialist)	1.022 (1.018–1.026)	1.026 (1.013–1.172)
Days of incapacity for work	1.012 (1.009–1.016)	1.002 (1.000–1.012)

* CI = confidence interval; OCD = outpatient-care diagnosis; HDD = hospital discharge diagnosis.

**Table 4:** Comparison of the predictive model performance.

C-Statistics (95% Confidence Interval)	Logistic Regression	Random Forest
Full list scenario	0.776 (0.768 to 0.785)	0.787 (0.777 to 0.792)
Core list scenario	0.793 (0.784 to 0.801)	0.800 (0.797 to 0.814)

**Table 5:** Further evaluation criteria for the predictive models based on the core list scenario.

Performance Metrics	Logistic Regression		Random Forest	
	High Risk *	Very High Risk *	High Risk *	Very High Risk *
Sensitivity	0.623	0.429	0.688	0.500
Specificity	0.815	0.911	0.781	0.889
Positive predictive value	0.309	0.391	0.295	0.375
Negative predictive value	0.942	0.923	0.949	0.930

* High-risk individuals = risk score 15–24%; very high risk individuals = risk score ≥ 25%.

## Data Availability

The data used for this paper were provided from the data warehouse of the OptiMedis AG. It comprises deidentified claims data from three different health insurance companies from one rural and one urban area in Germany. With specific permission and in an aggregated format, the data can be used for care improvement and research purposes, but publication or provision of the original raw data of individuals is contractually prohibited.

## Appendix A

**Table A1:** Regression coefficients of the independent variables in the logistic regression models for predicting ACSH in the full list model.

Independent Variable	Regression Coefficient	Sig.	Odds Ratio (OR)	Confidence Interval OR
Age (1)	0.199		1.220	0.978–1.521
Age (2)	0.536	**	1.709	1.313–2.224
Age (3)	0.677	***	1.971	1.397–2.781
Age (4)	0.762	***	2.142	1.475–3.111
Age (5)	0.964	***	2.621	1.816–3.783
Age (6)	1.029	***	2.630	1.817–3.808
Age (7)	1.020	***	2.797	1.926–4.064
Age (8)	1.205	***	2.775	1.921–4.007
Age (9)	1.205	***	3.338	2.334–4.773
Age (10)	1.395	***	4.034	2.832–5.746
Age (11)	1.473	***	4.364	3.062–6.219
Age (12)	1.830	***	6.236	4.301–9.041
Age (13)	2.070	***	7.927	5.454–11.522
Age (14)	2.087	***	8.064	5.563–11.690
Age (15)	2.311	***	10.080	6.967–14.583
Female	−0.191	***	0.826	0.800–0.853
Insurance status “employed”	0.037		1.038	0.886–1.246
Insurance status “pensioner”	0.160		1.173	0.966–1.373
Insurance status “child <18 years”	−0.469		0.612	0.310–1.309
Insurance status “child 18–25 years”	−0.018		0.982	0.640–1.502
Insurance status “unemployed”	0.062		1.064	0.780–1.456
Insurance status “other”	0.258		1.294	0.887–2.027
Days of incapacity for work	0.012	***	1.012	1.009–1.016
No. of outpatient visits (GP)	0.025		1.025	0.780–1.344
No. of outpatient visits (specialist)	0.022	***	1.022	1.018–1.026
No. of hospital stays	0.159	***	1.172	1.125–1.244
No. of ACSH	0.180	***	1.197	1.104–1.308
Days of hospital stays	−0.004		0.996	0.604–1.650
Drug count	0.049	***	1.050	1.036–1.064
Polypharmacy measure	−0.006		0.994	0.652–1.514
Multimorbidity score (Charlson index)	0.031		1.031	0.886–1.283
Long-term care level (1)	0.228		1.257	0.991–1.606
Long-term care level (2)	0.288	***	1.333	1.126–1.580
Long-term care level (3)	0.283	***	1.328	1.119–1.575
Days in long-term care level	0.001	***	1.000	1.000–1.001
DMP—Coronary Heart Disease	−0.140		0.869	0.534–1.356
DMP—Asthma	0.222		1.248	0.910–1.711
DMP—Type 2 Diabetes	0.683		1.976	0.842–2.827
DMP—COPD	0.575		1.755	0.635–4.105
OCD—Heart failure	−0.110		0.896	0.713–1.150
OCD—Other diseases of the circulation system	0.191	***	1.211	1.103–1.332
OCD—Bronchitis and COPD	0.002		1.002	0.848–1.184
OCD—Influenza and pneumonia	0.221	*	1.247	0.981–1.504
OCD—Essential hypertension	−0.009		0.991	0.804–1.174
OCD—Ear nose throat infections	0.124	***	1.132	1.058–1.173
OCD—Ischemic heart disease	0.042		1.043	0.829–1.345
OCD—Depressive disorders	0.099	**	1.104	1.016–1.213
OCD—Gastroenteritis and other diseases of intestines	0.063		1.065	0.902–1.258
OCD—Mental and behavioral disorders due to use of alcohol or opioids	0.914	***	2.520	1.799–3.244
OCD—Diabetes mellitus	0.159	*	1.172	1.019–1.406
OCD—Back pain (dorsopathies)	0.020		1.020	0.857–1.213
OCD—Other avoidable mental and behavioral disorders	0.085	*	1.089	1.030–1.314
OCD—Diseases of urinary system	0.041		1.042	0.717–1.656
OCD—Gonarthrosis (arthrosis of knee)	0.096	*	1.101	1.010–1.230
OCD—Intestinal infectious diseases	−0.074		0.928	0.804–1.091
OCD—Diseases of the eye	0.034		1.035	0.912–1.193
OCD—Soft tissue disorders	0.091		1.095	0.688–1.828
OCD—Melanoma and other malignant neoplasms of skin	0.040	*	1.041	1.005–1.088
OCD—Diseases of the skin and subcutaneous tissue	−0.099	**	0.905	0.885–0.918
OCD—Sleep disorders	0.061		1.063	0.909–1.245
OCD—Metabolic disorders	0.002		1.002	0.798–1.262
OCD—Migraine and headache syndromes	0.089		1.093	0.811–1.501
OCD—Gastritis and duodenitis	0.062		1.064	0.820–1.384
OCD—Thyroid disorder	0.055	*	1.056	1.008–1.419
OCD—Malnutrition and nutritional deficiencies	0.069		1.071	0.839–1.366
OCD—Dental diseases	0.050		1.051	0.731–1.570
OCD—Alcoholic liver disease	0.884	**	2.418	1.120–4.490
OCD—Asthma	0.606		1.791	0.671–4.141
OCD—Convulsions, not elsewhere classified	0.007		1.007	0.600–1.740
OCD—Maternal disorders related to pregnancy	1.946	***	7.079	4.874–10.281
OCD—Diseases of male genital organs	0.041		1.042	0.903–1.217
OCD—Other polyneuropathies	−0.022		0.978	0.829–1.133
OCD—Inflammatory diseases of female pelvic organs and disorders of female genital tract	0.052		1.053	0.736–1.558
OCD—Obesity	0.069		1.071	0.724–1.587
OCD—Decubitus ulcer and pressure area	0.046		1.047	0.545–2.429
OCD—Dementia	0.029		1.029	0.885–1.241
OCD—Avoidable infectious and parasitic diseases	−0.031		0.970	0.829–1.133
OCD—Perforated, bleeding ulcer	0.038		1.039	0.693–1.781
HDD—Heart failure	0.223	*	1.249	0.991–1.606
HDD—Other diseases of the circulation system	0.042		1.043	0.679–1.769
HDD—Bronchitis and COPD	0.108		1.114	0.722–1.716
HDD—Influenza and pneumonia	0.233		1.262	0.927–1.450
HDD—Essential hypertension	0.196	**	1.216	1.022–1.348
HDD—Ear nose throat infections	0.016		1.016	0.732–1.408
HDD—Ischemic heart disease	0.050		1.051	0.549–2.433
HDD—Depressive disorders	0.122	*	1.130	0.943–1.306
HDD—Gastroenteritis and other diseases of intestines	0.535		1.707	0.902–2.869
HDD—Mental and behavioral disorders due to use of alcohol or opioids	0.670	*	1.962	1.721–2.332
HDD—Diabetes mellitus	−0.019		0.981	0.814–1.183
HDD—Back pain (dorsopathies)	0.231		1.260	0.758–2.642
HDD—Other avoidable mental and behavioral disorders	0.122		1.130	0.912–1.400
HDD—Diseases of urinary system	0.007		1.007	0.874–1.161
HDD—Gonarthrosis (arthrosis of knee)	−0.021		0.979	0.647–1.032
HDD—Intestinal infectious diseases	0.070		1.073	0.907–1.269
HDD—Diseases of the eye	−0.007		0.993	0.599–1.645
HDD—Soft tissue disorders	−0.030		0.971	0.713–1.150
HDD—Melanoma and other malignant neoplasms of skin	0.128		1.136	0.876–1.476
HDD—Diseases of the skin and subcutaneous tissue	0.128		1.136	0.970–1.332
HDD—Sleep disorders	0.228		1.256	0.972–1.648
HDD—Metabolic disorders	0.142	*	1.152	1.010–1.506
HDD—Migraine and headache syndromes	0.121		1.129	0.984–1.295
HDD—Gastritis and duodenitis	0.044		1.045	0.787–1.441
HDD—Thyroid disorder	0.046	*	1.047	1.008–1.349
HDD—Malnutrition and nutritional deficiencies	0.064		1.066	0.861–1.321
HDD—Dental diseases	0.015		1.015	0.771–1.335
HDD—Alcoholic liver disease	0.109		1.115	0.937–1.327
HDD—Asthma	0.215		1.240	0.845–1.433
HDD—Convulsions, not elsewhere classified	0.108		1.114	0.950–1.307
HDD—Maternal disorders related to pregnancy	−0.112		0.894	0.594–1.398
HDD—Diseases of male genital organs	0.005		1.005	0.723–1.413
HDD—Other polyneuropathies	0.223		1.249	0.965–1.641
HDD—Inflammatory diseases of female pelvic organs and disorders of female genital tract	0.007		1.007	0.857–1.185
HDD—Obesity	0.106		1.111	0.898–1.374
HDD—Decubitus ulcer and pressure area	0.026		1.026	0.751–1.428
HDD—Dementia	0.010		1.010	0.832–1.229
HDD—Avoidable infectious and parasitic diseases	−0.096		0.908	0.244–3.116
HDD—Perforated, bleeding ulcer	0.038		1.039	0.712–1.711
Constant	−3.437	***	0.002	

* *p* < 0.1; ** *p* < 0.05; *** *p* < 0.01; OCD = outpatient-care diagnosis; HDD = hospital discharge diagnosis; DMP = Disease management program.

**Table A2:** Regression coefficients of the independent variables in the logistic regression models for predicting ACSH in the core list model.

Independent Variable	Regression Coefficient	Sig.	Odds Ratio (OR)	Confidence Interval OR
Age (1)	0.985	***	2.756	1.730–4.389
Age (2)	1.649	***	6.457	4.025–10.356
Age (3)	1.683	***	6.746	3.754–12.121
Age (4)	1.635	***	6.340	3.343–12.023
Age (5)	1.903	***	8.947	4.769–16.784
Age (6)	2.029	***	10.506	5.606–19.691
Age (7)	2.108	***	11.634	6.202–21.825
Age (8)	2.234	***	13.670	7.342–25.451
Age (9)	2.409	***	17.096	9.248–31.603
Age (10)	2.609	***	22.105	11.996–40.732
Age (11)	2.638	***	22.946	12.446–42.303
Age (12)	2.940	***	33.796	18.155–62.912
Age (13)	3.146	***	43.993	23.601–82.002
Age (14)	3.183	***	46.109	24.777–85.807
Age (15)	3.363	***	58.097	31.255–107.989
Female	−0.199	***	0.819	0.773–0.847
Insurance status “employed”	−0.111		0.895	0.713–1.747
Insurance status “pensioner”	0.167		1.182	0.754–1.435
Insurance status “child <18 years”	−0.759		0.640	0.532–1.719
Insurance status “child 18–25 years”	−0.017		0.983	0.976–1.189
Insurance status “unemployed”	0.073		1.076	0.756–1.715
Insurance status “other”	0.311		1.364	0.691–2.444
Days of incapacity for work	0.002	***	1.002	1.000–1.012
No. of outpatient visits (GP)	0.029		1.030	0.767–1.584
No. of outpatient visits (specialist)	0.025	***	1.026	1.013–1.172
No. of hospital stays	0.162	***	1.175	1.109–1.269
No. of ACSH	0.182	***	1.199	1.114–1.320
Days of hospital stays	−0.010		0.990	0.732–1.307
Drug count	0.057	***	1.059	1.016–1.239
Polypharmacy measure	−0.009		0.991	0.798–2.163
Multimorbidity score (Charlson index)	0.034		1.035	0.808–1.425
Long-term care level (1)	0.286	**	1.331	1.049–1.688
Long-term care level (2)	0.291	***	1.338	1.131–1.582
Long-term care level (3)	0.227	***	1.255	1.058–1.488
Days in long-term care level	0.001	***	1.000	1.000–1.001
DMP—Coronary Heart Disease	−0.124		0.883	0.662–1.201
DMP—Asthma	0.243		1.272	0.896–1.869
DMP—Type 2 Diabetes	0.829		2.329	0.732–3.433
DMP–COPD	0.821		2.278	0.823–5.859
OCD—Heart failure	−0.110		0.896	0.580–1.150
OCD—Other diseases of the circulation system	0.259	***	1.295	1.243–1.807
OCD—Bronchitis and COPD	0.003		1.003	0.820–1.504
OCD—Influenza and pneumonia	0.170		1.185	0.943–1.356
OCD—Essential hypertension	0.019		1.019	0.776–1.488
OCD—Ear nose throat infections	0.143	***	1.153	1.083–1.353
OCD—Ischemic heart disease	0.078		1.080	0.977–2.515
OCD—Depressive disorders	0.094	**	1.098	1.041–1.249
OCD—Gastroenteritis and other diseases of intestines	0.074		1.077	0.985–1.472
OCD—Mental and behavioral disorders due to use of alcohol or opioids	0.997	***	2.706	1.963–3.539
OCD—Diabetes mellitus	0.175	*	1.192	1.114–1.551
OCD—Back pain (dorsopathies)	0.026		1.027	0.843–1.598
OCD—Other avoidable mental and behavioral disorders	0.089	*	1.093	1.009–1.382
OCD—Diseases of urinary system	0.034		1.035	0.984–1.389
OCD—Gonarthrosis (arthrosis of knee)	0.125	*	1.133	1.069–1.608
OCD—Intestinal infectious diseases	−0.069		0.933	0.854–1.223
OCD—Diseases of the eye	0.011		1.011	0.918–1.391
OCD—Soft tissue disorders	0.110		1.116	0.492–2.210
OCD—Melanoma and other malignant neoplasms of skin	0.056	*	1.058	1.021–1.408
OCD—Diseases of the skin and subcutaneous tissue	−0.106	**	0.900	0.830–0.969
OCD—Sleep disorders	0.073		1.076	0.918–1.488
OCD—Metabolic disorders	−0.035		0.966	0.744–1.439
OCD—Migraine and headache syndromes	0.078		1.080	0.850–1.319
OCD—Gastritis and duodenitis	0.074		1.077	0.713–1.663
OCD—Thyroid disorder	0.096	*	1.101	1.018–2.483
OCD—Malnutrition and nutritional deficiencies	0.080		1.082	0.826–1.591
OCD—Dental diseases	0.051		1.052	0.811–1.592
OCD—Alcoholic liver disease	0.894	**	2.464	1.192–4.331
OCD—Asthma	0.624		1.813	0.873–4.267
OCD—Convulsions, not elsewhere classified	0.004		1.004	0.815–1.263
OCD—Maternal disorders related to pregnancy	1.569	***	4.881	3.930–8.291
OCD—Diseases of male genital organs	0.038		1.039	0.791–1.126
OCD—Other polyneuropathies	−0.008		0.992	0.874–1.408
OCD—Inflammatory diseases of female pelvic organs and disorders of female genital tract	0.013		1.013	0.771–1.482
OCD—Obesity	0.097		1.102	0.698–2.229
OCD—Decubitus ulcer and pressure area	0.046		1.047	0.741–2.412
OCD—Dementia	0.035		1.036	0.836–1.514
OCD—Avoidable infectious and parasitic diseases	−0.024		0.976	0.892–1.288
OCD—Perforated, bleeding ulcer	0.032		1.033	0.601–1.490
HDD—Heart failure	0.218	*	1.244	1.003–1.573
HDD—Other diseases of the circulation system	0.023		1.023	0.629–1.978
HDD—Bronchitis and COPD	0.105		1.110	0.720–1.661
HDD—Influenza and pneumonia	0.211		1.236	0.971–1.311
HDD—Essential hypertension	0.160	**	1.173	1.047–1.300
HDD—Ear nose throat infections	0.044		1.045	0.804–3.888
HDD—Ischemic heart disease	0.028		1.029	0.919–1.380
HDD—Depressive disorders	0.120	*	1.128	1.077–1.983
HDD—Gastroenteritis and other diseases of intestines	0.814		2.238	0.906–4.367
HDD—Mental and behavioral disorders due to use of alcohol or opioids	0.964	*	2.622	1.133–3.356
HDD—Diabetes mellitus	0.011		1.011	0.930–1.686
HDD—Back pain (dorsopathies)	0.221		1.247	0.896–2.530
HDD—Other avoidable mental and behavioral disorders	0.130		1.138	0.964–1.488
HDD—Diseases of urinary system	0.009		1.009	0.872–1.303
HDD—Gonarthrosis (arthrosis of knee)	−0.091		0.912	0.754–1.455
HDD—Intestinal infectious diseases	0.092		1.096	0.963–1.660
HDD—Diseases of the eye	0.031		1.032	0.979–1.339
HDD—Soft tissue disorders	−0.003		0.997	0.830–1.512
HDD—Melanoma and other malignant neoplasms of skin	0.105		1.111	0.831–1.214
HDD—Diseases of the skin and subcutaneous tissue	0.189		1.209	0.776–1.962
HDD—Sleep disorders	0.125		1.133	0.930–1.503
HDD—Metabolic disorders	0.139		1.149	0.922–1.479
HDD—Migraine and headache syndromes	0.117		1.125	0.927–1.254
HDD—Gastritis and duodenitis	0.015		1.015	0.887–1.496
HDD—Thyroid disorder	0.044	*	1.045	1.005–1.303
HDD—Malnutrition and nutritional deficiencies	0.072		1.075	0.886–1.477
HDD—Dental diseases	0.028		1.029	0.942–2.481
HDD—Alcoholic liver disease	0.114		1.120	0.976–1.390
HDD—Asthma	0.198		1.219	0.838–1.433
HDD—Convulsions, not elsewhere classified	0.122	*	1.130	1.005–1.481
HDD—Maternal disorders related to pregnancy	−0.109		0.897	0.644–1.157
HDD—Diseases of male genital organs	0.017		1.017	0.831–1.745
HDD—Other polyneuropathies	0.114		1.120	0.982–1.836
HDD—Inflammatory diseases of female pelvic organs and disorders of female genital tract	0.028		1.029	0.312–2.728
HDD—Obesity	0.115		1.123	0.953–1.494
HDD—Decubitus ulcer and pressure area	0.019		1.019	0.705–1.418
HDD—Dementia	0.017		1.017	0.835–1.313
HDD—Avoidable infectious and parasitic diseases	−0.091		0.912	0.791–1.642
HDD—Perforated, bleeding ulcer	0.042		1.043	0.720–1.899
Constant	−3.721	***	1.000	

* *p* < 0.1; ** *p* < 0.05; *** *p* < 0.01; OCD = outpatient-care diagnosis; HDD = hospital discharge diagnosis; DMP = Disease management program.
